# Natural variation in *ZmNAC087* contributes to total root length regulation in maize seedlings under salt stress

**DOI:** 10.1186/s12870-023-04393-7

**Published:** 2023-08-14

**Authors:** Xiaomin Zhang, Houmiao Wang, Mengling Yang, Runxiao Liu, Xin Zhang, Zhongtao Jia, Pengcheng Li

**Affiliations:** 1 State Key Laboratory of Crop Stress Adaptation and Improvement, Academy for Advanced Interdisciplinary Studies, School of Life Sciences, Henan University, Kaifeng, 475004 China; 2 Sanya Institute, Henan University, Sanya, 572025 China; 3 Jiangsu Key Laboratory of Crop Genetics and Physiology, Key Laboratory of Plant Functional Genomics of the Ministry of Education, Jiangsu Key Laboratory of Crop Genomics and Molecular Breeding, Yangzhou University, Yangzhou, 225009 China; 4 Jiangsu Co-Innovation Center for Modern Production Technology of Grain Crops, Yangzhou University, Yangzhou, 225009 China; 5 State Key Laboratory of Nutrient Use and Management (SKL-NUM), College of Resources and Environmental Sciences, National Academy of Agriculture Green Development, China Agricultural University, Beijing, 100193 China

**Keywords:** Maize (*Zea mays*), Salt stress, Total root length, Natural variation, Domestication selection, *ZmNAC087*

## Abstract

Soil salinity poses a significant challenge to crop growth and productivity, particularly affecting the root system, which is vital for water and nutrient uptake. To identify genetic factors that influence root elongation in stressful environments, we conducted a genome-wide association study (GWAS) to investigate the natural variation associated with total root length (TRL) under salt stress and normal conditions in maize seedlings. Our study identified 69 genetic variants associated with 38 candidate genes, among which a specific single nucleotide polymorphism (SNP) in *ZmNAC087* was significantly associated with TRL under salt stress. Transient expression and transactivation assays revealed that *ZmNAC087* encodes a nuclear-localized protein with transactivation activity. Further candidate gene association analysis showed that non-coding variations in *ZmNAC087* promoter contribute to differential *ZmNAC087* expression among maize inbred lines, potentially influencing the variation in salt-regulated TRL. In addition, through nucleotide diversity analysis, neutrality tests, and coalescent simulation, we demonstrated that *ZmNAC087* underwent selection during maize domestication and improvement. These findings highlight the significance of natural variation in *ZmNAC087*, particularly the favorable allele, in maize salt tolerance, providing theoretical basis and valuable genetic resources for the development of salt-tolerant maize germplasm.

**Supplementary Information:**

The online version contains supplementary material available at 10.1186/s12870-023-04393-7.

## Background

Soil salinity is a major constraint on global crop production and poses a significant threat to sustainable agriculture and food supply [1, 2]. High soil salt levels, resulting from sodium chloride (NaCl) irrigation and exacerbated by climate change, can cause ion toxicity, osmotic stress, and nutrient imbalances in plants [3–5]. These stressors disrupt plant metabolism, oxidation–reduction systems, and photosynthesis, leading to stunted growth and even plant mortality [6, 7]. As a staple crop that contributes to global calorie consumption [8], Maize (*Zea may*s) is particularly susceptible to salt stress [9, 10], limiting its yield potential. Therefore, it is crucial to explore genetic factors underlying salt tolerance and generate salt-tolerant maize varieties to ensure global food security.

Salt tolerance is a complex and quantitative trait in plants. Sodium exclusion has been extensively studied as a salt tolerance-related quantitative trait locus (QTL). Multiple QTLs associated with sodium concentration have been identified in various crops, such as rice and wheat [11–13]. For example, *Shoot K* ^+^ *Content 1* (SKC1) in rice and *Na* ^+^ *Exclusion 1* and* 2* (*Nax1 and 2*) in wheat function as Na^+^-selective transporters that improve salt tolerance by regulating the ratio of K^+^/Na^+^ [14–16]. The maize *Na* ^+^ *content 2* (*ZmNC2*) encodes an ion transporter that belongs to the HAK family and mediates Na^+^ exclusion from shoots, thereby improving salt tolerance [17]. These QTLs play critical roles in maintaining a balance between potassium and sodium ions to improve salt tolerance. Moreover, the SOS signaling pathway has been implicated in facilitating sodium efflux in maize roots [13]. Therefore, understanding natural variations in the SOS signaling pathway and its regulatory factors is essential for deciphering the observed differences in salt tolerance among maize inbred lines.

Root development is a key phenotype used to evaluate salt tolerance [18, 19]. In salinized soil, a significant influx of Na^+^ ions occurs towards the plant roots, causing osmotic stress that negatively impacts multiple aspects of the rhizosphere, including inhibition of root elongation, reduction in root turgor pressure, and impairment of water and nutrient absorption from the soil [20, 21]. Architecture of the root system therefore undergoes adjustments to optimize resource exploration in response to these adverse changes [22, 23]. In the model plant *Arabidopsis*, the root system consists of a primary root (PR) and postembryonic lateral roots (LRs), which can further branch into higher-order LRs and elevated salinity levels have been shown to hinder the growth of both the PR and LRs [24, 25]. By contrast, Maize possesses a more sophisticated root system that consists of a primary root, seminal roots, and embryonic adventitious roots (crown and brace roots) [26, 27]. These roots are formed during distinct developmental stages and exhibit remarkable branching capabilities, enabling extensive lateral root development and efficient exploration of the soil volume [28, 29]. Maize root growth is generally hindered by salt stress, with different root types exhibiting differential responses [3, 30]. Total root length (TRL) is an excellent target for improving salt tolerance due to its direct impact on water and nutrient uptake [18, 31]. Improving TRL can significantly enhance crop yield by facilitating efficient resource acquisition. Identification of the genetic components and natural variations that influence root performance will provide theoretical guidance and genetic resource for breeding salt-tolerant maize varieties.

NAM-ATAF-CUC2 (NAC) transcription factors represent one of the largest and most specific families of transcription factors in plants. They play diverse roles in regulating biochemical and developmental pathways. According to whole genome characterization, there are 105 NAC members in *Arabidopsis*, 121 in rice, and 147 in maize [32, 33]. NAC genes have a conserved DNA-binding domain (NAC domain) in the N-terminal region, along with a highly variable DNA-activating domain in the C-terminal region, which may be crucial for their specific binding to genes involved in various processes such as root development [34], xylem formation [35], leaf senescence [36], nutrient remobilization [37], and abiotic stress responses [38]. The involvement of NAC transcription factors (TFs) in stress response has garnered significant attention. Notably, several NAC TFs, including *OsSNAC1*, *OsSNAC3*, and *OsONAC022*, have been identified as key regulators of drought and salt tolerance in rice [39–41]. Moreover, overexpression of the wheat *TaNAC071*-A, and maize *ZmSNAC1*, *ZmNAC049*, and *ZmNAC111* leads to significant improvements in drought tolerance [42–45]. However, the mechanisms underlying NAC-mediated stress tolerance remain poorly understood, partly due to the large number of NAC transcription factors present in plant genomes.

Maize is an ideal model for genome-wide association studies (GWAS) due to its extensive genetic diversity and rapid linkage disequilibrium (LD) decay [46]. Previous studies have demonstrated substantial variability in salt tolerance among maize inbred lines, suggesting the presence of salt-tolerant genetic variations that can be utilized for developing salt-resistant maize cultivars [4, 17]. In this study, we conducted a GWAS to identify the associations between natural variations in *ZmNAC087* and TRL under salt stress conditions. Additionally, we performed gene-based association analysis by re-sequencing *ZmNAC087* in 32 teosintes, 71 landraces, and 280 inbred lines. The objectives of our research were to identify natural variations associated with TRL under salt stress, investigate nucleotide diversity in *ZmNAC087* across teosinte and maize populations, and explore the contribution of *ZmNAC087* to maize domestication and improvement. These findings provide a theoretical basis for salt responses in maize and contribute to the development of genetic markers for enhancing salt tolerance.

## Results

### GWAS identifies *ZmNAC087* as a key gene for natural variation in total root length (TRL) under salt stress

**Fig. 1:**
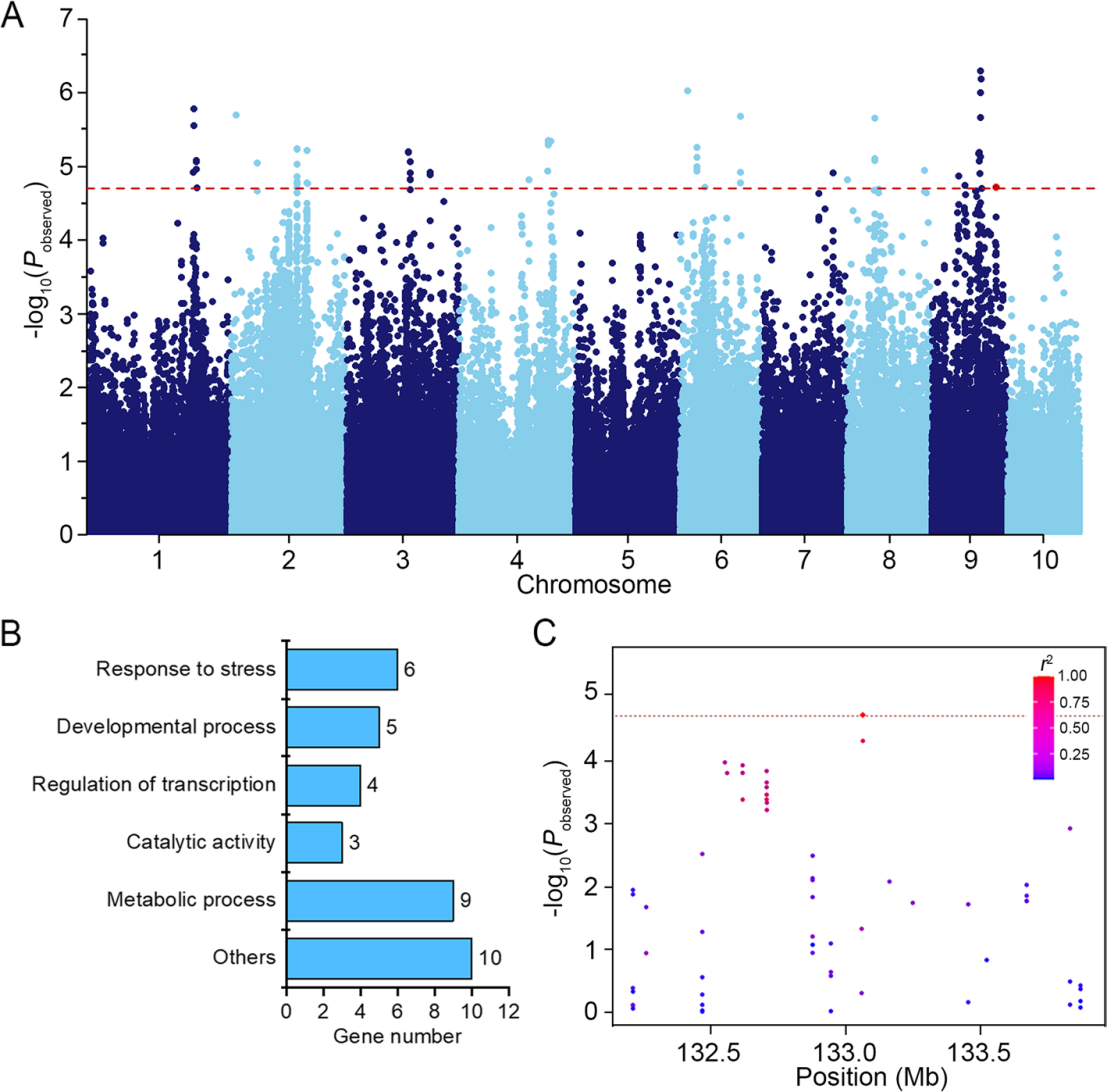
*ZmNAC087* is Associated with Natural Variations in Total Root Length (TRL) under Salt Stress. **A** Manhattan plot showing marker-trait associations for TRL under 100 mM NaCl. The red dotted line represents the significance threshold (-log_10_ *P* = 4.71). A significant SNP in *ZmNAC087* is highlighted in red. **B** Histogram showing the biological pathway associated with the 37 candidate genes identified by GWAS under normal and salt-stress conditions. **C** Local Manhattan plot showcasing the genomic region of *ZmNAC087* on chromosome 9. The plot focuses on the 1-Mb region surrounding the most significant SNP. The leading SNP is depicted with a red diamond and the remaining SNPs are indicated by colored dots based on their linkage disequilibrium (LD, *r* ^2^) with the leading SNP

In our study, we conducted a comprehensive evaluation of genetic variation in root architecture across 280 maize accessions under both normal and salt-stress conditions. Our observations revealed a wide range of phenotypic variations in TRL, spanning from 48.9 cm to 735.73 cm and 67.66 cm to 370.18 cm under normal and salt stress, respectively [31]. To further investigate the genetic loci associated with the variation in maize TRL, we performed a Genome-Wide Association Study (GWAS) using a mixed linear model (MLM), which was corrected for kinship (K) and population structure (Q) to mitigate false positives. We identified 66 markers that showed significant associations with TRL specifically under salt-stress conditions. These markers were further resolved to 36 candidate genes, which were distributed across all chromosomes except for Chromosomes 5 and 10. These candidate genes collectively explained approximately 51.0% of the observed phenotypic variation (Fig. 1A and Table S1). By contrast, only three significant associations were observed under the normal condition, leading to the identification of a single candidate gene (Fig. S1 and Table S1). Gene ontology analysis revealed that six of the candidate genes are associated with a biological pathway involved in plant stress response. The remaining genes were predicted to participate in various processes, including development, transcriptional regulation, catalytic activity, and metabolism (Fig. 1B and Table S1). A single nucleotide polymorphism (SNP) located in the GRMZM2G159500 (B73_V3) gene on chromosome 9 exhibited a specific linkage to TRL under salt stress (Fig. 1A, C, and Fig. S1). This gene encodes a member of the NAC transcription factor (TF) family and phylogenetic analysis based on amino acid sequences revealed its significant similarity to the Arabidopsis *NAC087* gene. It was therefore designated as *ZmNAC087* (Fig. S2).

### ZmNAC087 is localized in the nucleus and exhibits transactivation activity

**Fig. 2:**
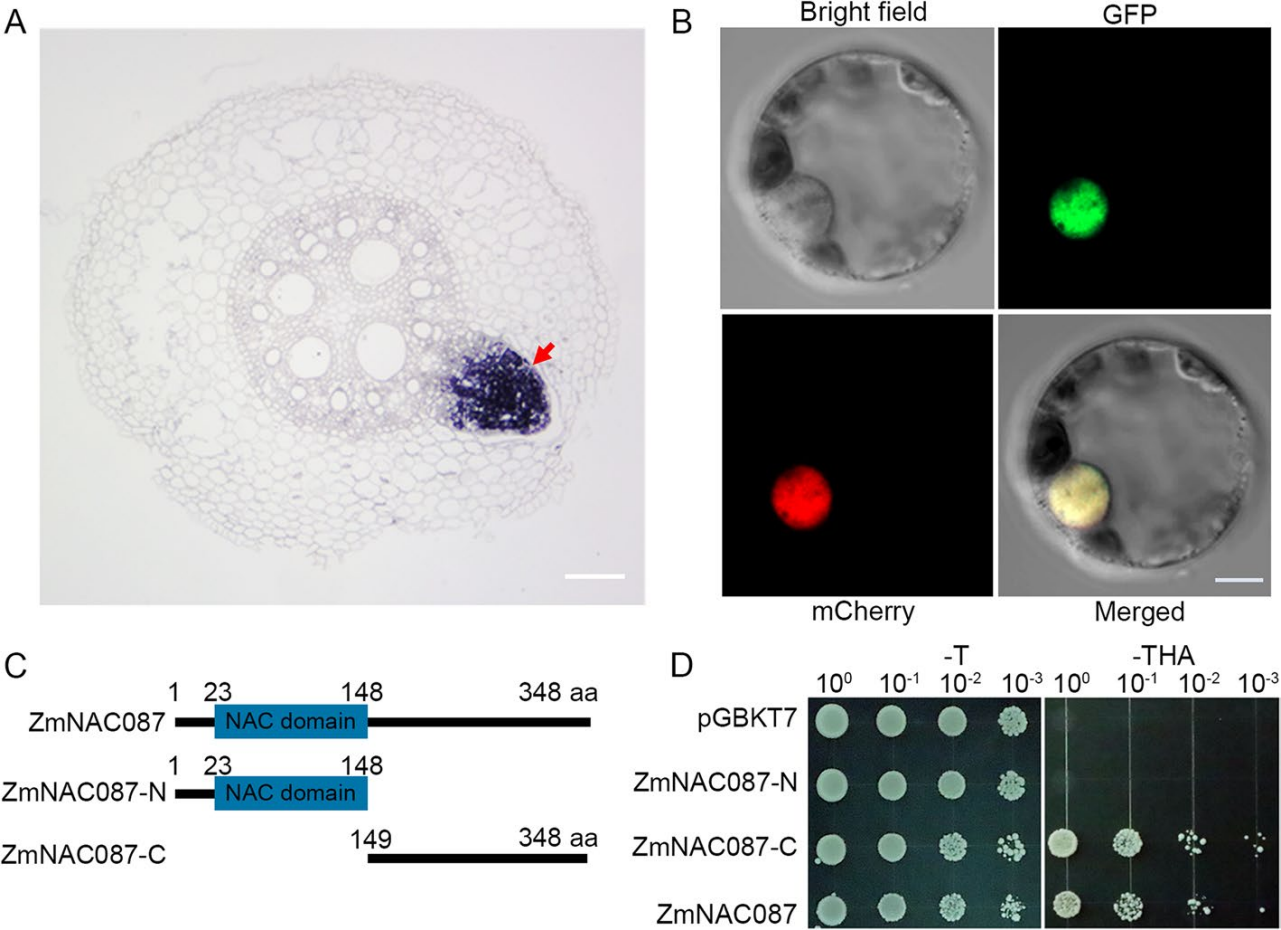
Tissue-specific Expression, Subcellular Localization, and Transactivation Activity of ZmNAC087. **A** Localization of ZmNAC087 in the cross-section of 7-day-old hydroponically grown roots of B73 was determined using a DIG-labeled RNA antisense probe. The scale bar represents 100 μm. **B** Maize protoplasts co-transfected with *Ubi:ZmNAC087-GFP* and *35S:H2B-mCherry* plasmids. H2B-mCherry served as a marker for nuclear localization. The scale bar represents 5 μm. **C** Schematic representation of *ZmNAC087* gene structure. **D** Assessment of ZmNAC087 transcriptional activation activity in yeast. The full-length coding sequence (1–348 aa, ZmNAC087), sequence of the N-terminus (1–148 aa, ZmNAC087-N), and sequence of the C-terminus (149–348 aa, ZmNAC087-C) were individually cloned into the pGBKT7 vector. Transformed yeast cells were grown on SD/–Trp and SD/–Trp–His–Ade media. The yeast concentrations were adjusted to an OD_600_ value of 0.1 and then diluted to 1/10, 1/100, and 1/1000 before incubating on the plates at 30 °C for 3 days

To gain insights into the role of *ZmNAC087* in regulating root growth under salt stress, we conducted in situ mRNA localization in B73 root tissues to determine its tissue-specific expression profile. *ZmNAC087* mRNA was predominantly expressed in the stele of the PR and LP primordium (Fig. 2A). To determine the subcellular localization of ZmNAC087, we fused green fluorescent protein (GFP) to its C-terminus in frame and transiently expressed the fusion protein in maize mesophyll protoplasts using a strong constitutive *ZmUbi* promoter. The nuclear marker H2B-mCherry [47] was co-expressed with ZmNAC087-GFP and green fluorescence for ZmNAC087-GFP was detected in the nucleus and showed perfect colocalization with H2B-mCherry (Fig. 2B), providing strong evidence for the specific nuclear localization of ZmNAC087-GFP.

Bioinformatic analysis predicted the presence of a conserved NAC domain in the N-terminus of ZmNAC087, whereas the C-terminus contains a transactivation domain. Although most NAC transcription factors act as activators, previous studies have shown that some members of this family can function as transcriptional repressors [35]. To investigate the biochemical function of ZmNAC087, we assessed its transactivation activity using a yeast activation system. The full-length coding sequence of *ZmNAC087* was fused with the GAL4 DNA-binding domain in the expression vector pGBKT7. Truncated versions of the protein, ZmNAC087-N (N-terminal half) and ZmNAC087-C (C-terminal half), were also generated to identify the essential domain for its transcriptional activation activity (Fig. 2C). Cells transformed with empty vectors and ZmNAC087-N showed no growth on synthetic dropout media that lacked tryptophan, histidine, and adenine. By contrast, cells transformed with ZmNAC087 or ZmNAC087-C displayed robust growth on this medium (Fig. 2D). These results confirm that ZmNAC087 functions as a transcriptional activator, and its transactivation activity is mediated by the C-terminal domain.

### Non-coding variations in *ZmNAC087* are associated with total root length (TRL) under salt stress

**Fig. 3:**
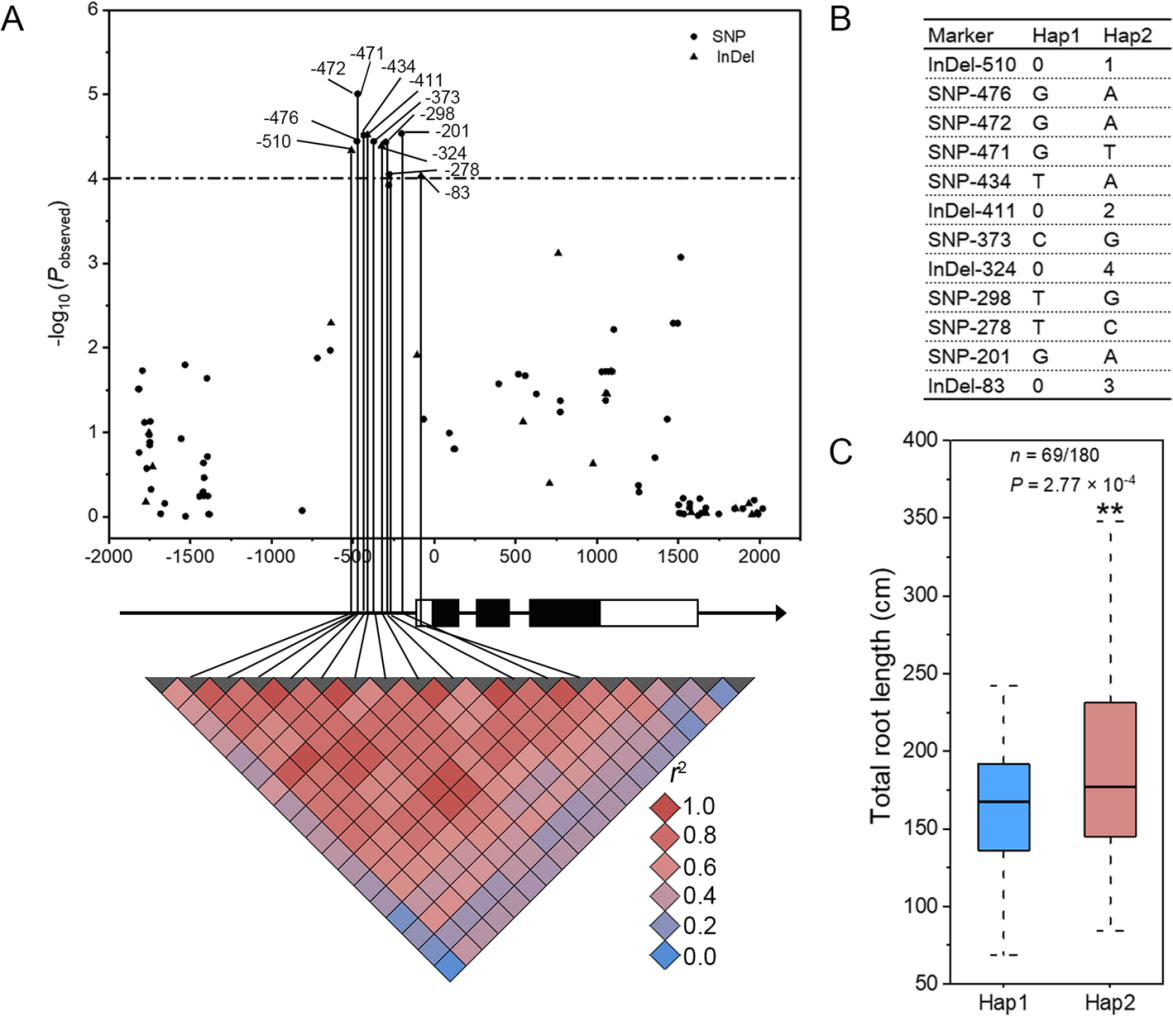
Association analysis of *ZmNAC087* genetic variations with maize total root length (TRL) under salt stress. **A** Association of *ZmNAC087* genetic variants with TRL under salt stress. The black dots and triangles represent SNPs and InDels, respectively. The diagram depicts the 3.8-kb *ZmNAC087* genomic region that includes the 1.8-kb promoter, 1.0-kb coding sequence, and 1.0-kb downstream sequence. The start codon (ATG) of *ZmNAC087* is indicated as " + 1". The exons and UTRs are depicted as filled and open boxes, respectively, and the introns and promoter region are represented by black lines. The *p*-values are presented on a -log_10_ scale. The twelve significant polymorphisms in strong LD within the promoter region are connected to the pairwise LD diagram with black lines. **B** Association of *ZmNAC087* with TRL. Two haplotype groups, Hap1 and Hap2, are determined based on the LD analysis. **C** Comparison of TRL of salt-grown maize inbred lines that carry different haplotypes. Statistical significance was determined using a two-tailed *t*-test (*n* = 69 for Hap1 and 180 for Hap2)

To gain a better understanding of the impact of *ZmNAC087* genetic variations on maize salt tolerance, we re-sequenced the 3.8-kb genomic sequence of *ZmNAC087* in 280 inbred lines and identified 80 SNPs and 21 InDels (Table S2). Through MLM-based candidate gene association analysis, we identified twelve significant polymorphisms in *ZmNAC087* promoter, namely InDel-510, SNP-476, SNP-472, SNP-471, SNP-434, InDel-411, SNP-373, InDel-324, SNP-298, SNP-278, SNP-201, and InDel-83 (P < 9.90 × 10^−5^) (Fig. 3A). The two most significantly associated SNPs, SNP-472 and SNP-471, which are located at positions 472-bp and 471-bp upstream of the *ZmNAC087* start codon (ATG), explained the highest amount of phenotypic variation (r^2^ = 5.87%). The twelve significantly associated SNPs/InDels displayed strong linkage disequilibrium (LD) and enabled the classification of the maize population into two distinct haplotype groups, Hap1 and Hap2 (Fig. 3A-B). Inbred lines carrying the Hap2 haplotype displayed longer TRL on average compared with those carrying Hap1 under salt stress, indicating that Hap2 confers greater salt tolerance (Fig. 3C). This suggests that non-coding variations in *ZmNAC087* promoter significantly influence maize root growth under salt stress.

**Fig. 4:**
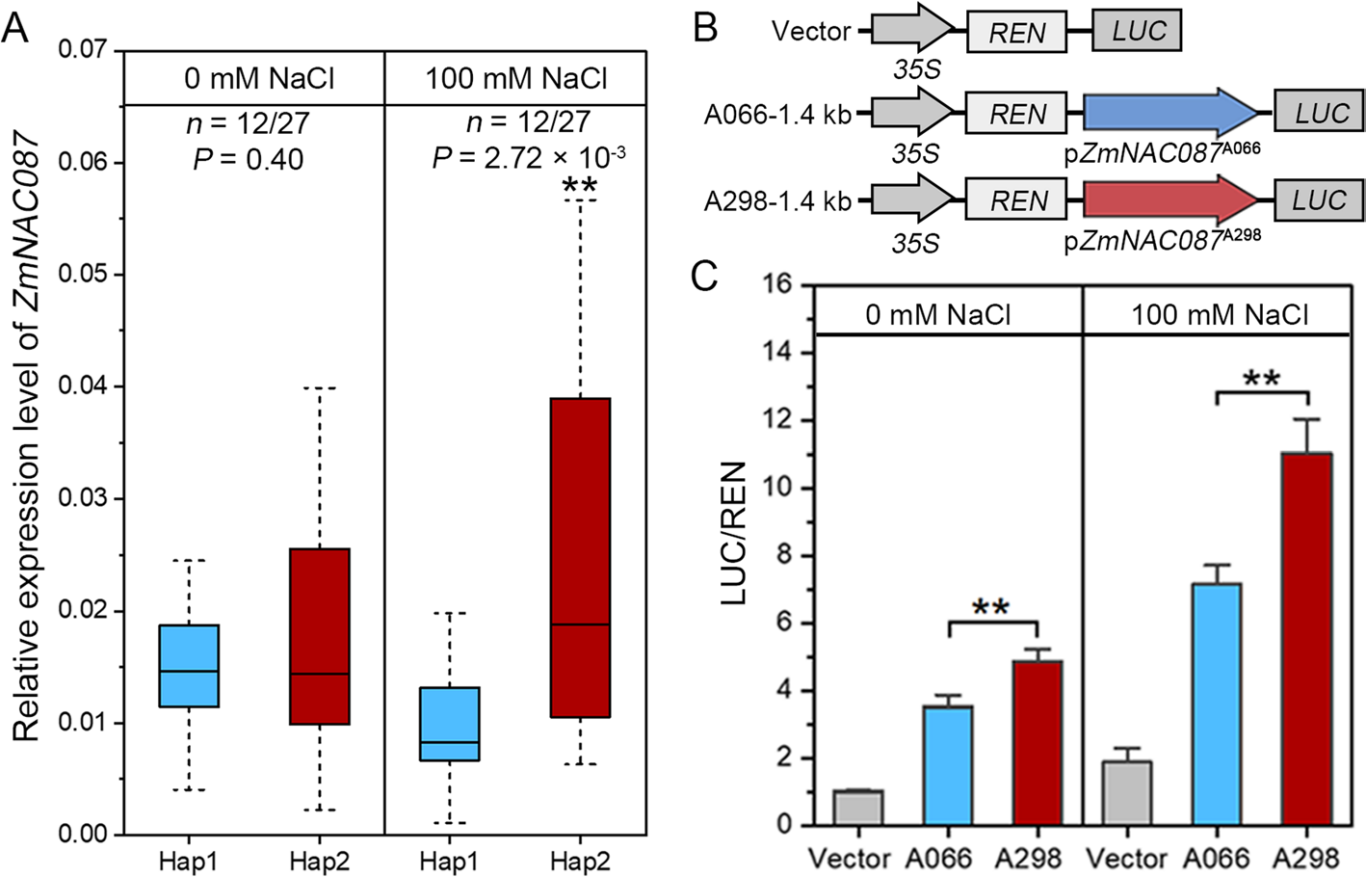
Association between genetic variations in *ZmNAC087* promoter with TRL. **A** Box plot showing the distribution of relative expression levels of *ZmNAC087* in 10-day-old hydroponically-grown maize roots under normal and 100 mM NaCl. The *ZmUbi2* gene served as the endogenous control. The statistical significance of the data was determined using a two-tailed *t*-test, **p < 0.01. **B** Schematic diagram showing the structure of the *ZmNAC087* ^A066^ and *ZmNAC087*.^A298^ vectors. **C** Transient expression assay using *ZmNAC087* promoter fragments that contained Hap1 and Hap2. The transfection efficiency was evaluated using *35S:Renilla luciferase* as a positive control. Statistical significance was determined using a two-tailed *t*-test: **p < 0.01

Based on the presence of twelve significant polymorphisms in the promoter region, the differential expression of *ZmNAC087* may account for the natural variation in salt tolerance among maize inbred lines. To test this hypothesis, the transcript level of *ZmNAC087* was measured in 12 salt-sensitive (which carried Hap1) and 27 salt-tolerant (which carried Hap2) lines under normal and salt-stressed conditions. No difference in *ZmNAC087* transcript level was detected between the two groups under normal conditions (Fig. 4A), by contrast, the transcript level of *ZmNAC087* was significantly higher in Hap2 than in Hap1 varieties, indicating a strong association between *ZmNAC087* expression and salt tolerance in the maize inbred lines tested. To ascertain whether *ZmNAC087* is differentially expressed due to the natural variation in the promoter region, we cloned ~ 1.4 kb promoter fragments from salt-sensitive A066 (Hap1) and salt-tolerant inbred lines A298 (Hap2) upstream of a LUC (luciferase) to compare their promoter activities (Fig. 4B). We found that irrespective of salt treatment, LUC expression driven by *ZmNAC087* ^A298^ was much stronger than that of *ZmNAC087* ^A066^ (Fig. 4C), suggesting that *ZmNAC087* ^A298^ has higher promoter activity compared to *ZmNAC087* ^A066^. Together, these results serve as strong evidence that natural variations in *ZmNAC087* promoter are responsible for differential *ZmNAC087* expression and TRL in maize inbred lines under salt stress.

### Sequence polymorphisms and selection of *ZmNAC087* in ancient and modern maize germplasm populations

We then investigated the evolutionary history of *ZmNAC087* during maize domestication and improvement by amplifying and re-sequencing a 3.8-kb genomic sequence of *ZmNAC087* in 280 maize inbred lines, 71 maize landraces, and 32 teosintes. Approximately 5.7-kb nucleotides sites were produced after alignment. In this 5.7-kb region, 2573-bp sites are upstream, 243-bp sites are in the 5′-UTR, 1624-bp sites are in the coding region, 591-bp sites are in the 3′-UTR, and 670-bp sites are in the downstream (Table 1). A total of 574 polymorphic sites, including 370 SNPs and 204 InDels, were identified, with an average frequency of 0.065 and 0.195, respectively. The average length of InDels varied across different genomic regions, with the upstream region exhibiting the greatest average length (~ 10 bp) and the 5'-UTR showing the smallest (4 bp). The average length of InDels across the entire genomic region was 6.8 bp. Further analysis revealed that SNPs and InDels were most abundant in the 3′-UTR and downstream regions, with frequencies of 0.13 (1 per 7.69 bp) and 0.254 (1 per 3.94 bp), respectively. The overall nucleotide diversity of *ZmNAC087* was 10.21 (π × 1000), and the 5′-UTR exhibited a significantly lower diversity compared with the other segments analyzed in *ZmNAC087*.

**Table 1:** Summary of sequence variation parameters in *ZmNAC087*

Parameters	Upstream region	5' UTR	Coding region	3' UTR	Downstream region	Entire Region
Number of nucleotide sites	2573	243	1624	591	670	5692
Total number of sequence variants identified	160	18	180	106	111	574
Frequency of each sequence variant	0.062	0.074	0.111	0.179	0.166	0.101
Number of nucleotide substitutions (bp)	91	10	123	77	69	370
Frequency of polymorphic sites (per bp)	0.035	0.041	0.076	0.130	0.103	0.065
Number of InDel events	69	8	57	29	42	204
Number of InDels	542	29	217	101	170	1112
Average length of InDels	10.232	4.0	5.439	4.621	4.976	6.809
Frequency of InDels (per bp)	0.211	0.119	0.134	0.171	0.254	0.195
π × 1000	8.85	2.91	10.06	15.92	8.68	10.21
θ × 1000	22.81	11.19	17.27	27.26	29.83	21.51
Tajima’s D	-1.804*	-1.606	-1.249	-1.216	-2.056*	-1.609
Fu and Li’s D*	-4.729**	-2.889*	-5.447**	-5.141**	-8.448**	-7.228**
Fu and Li’s F*	-3.929**	-2.909*	-3.938**	-3.874**	-6.463**	-4.933**

SNP refers to single nucleotide polymorphism, InDel refers to insertion-deletion. UTR represents untranslated region

* and ** indicate statistical significance at the *p* < 0.05 and *p* < 0.01 level, respectively

**Fig. 5:**
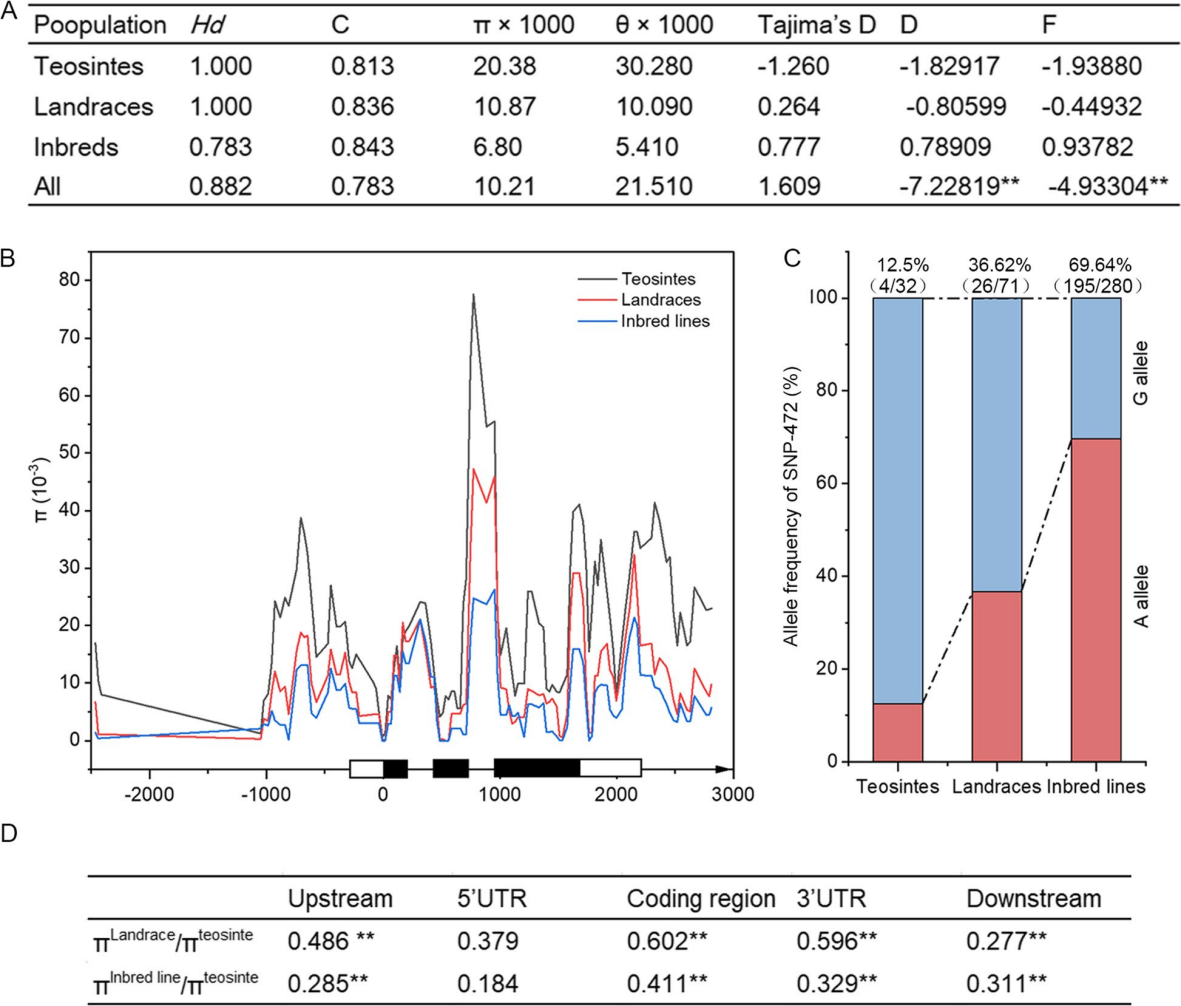
Gene diversity, allele frequencies, and nucleotide diversity of *ZmNAC087* in teosintes, landraces, and maize inbred lines. **A** Nucleotide polymorphisms in *ZmNAC087* and results of the neutrality test. Parameters include haplotype diversity (Hd), density of single nucleotide polymorphisms (Dens), sequence conservation (**C**), and Fu and Li's D* and F* values. Statistical significance is indicated by asterisks (*p < 0.05, **p < 0.01). **B** Nucleotide diversity (π) assay of the *ZmNAC087* genomic region in teosintes, landraces, and maize inbred lines. The schematic diagram illustrates the genomic structure of *ZmNAC087*, including the promoter region, exons (filled boxes), introns (black lines), and the 5'- and 3'-UTRs (open boxes). **C** Frequencies of the SNP-472 A and G alleles in teosintes, landraces, and inbred lines. The number in parentheses represents the count of accessions carrying each allele in each population. **D** The relative ratio of nucleotide diversity (π) in landraces and inbred lines compared with teosintes. The asterisk-labeled values indicate significant deviations from the expected results under a neutral maize domestication bottleneck in the corresponding regions (**, *p* < 0.01)

To examine whether *ZmNAC087* was selected during maize evolution, nucleotide diversity and sequence conservation (C) were compared among the three populations. This analysis showed an overall C value of 0.783 and a nucleotide diversity of 10.21 (π × 1000) for *ZmNAC087* across all three populations (Fig. 5A). Notably, the inbred lines (π × 1000_I_ = 6.80) exhibited lower nucleotide diversity compared with landraces (π × 1000_L_ = 10.87) and teosintes (π × 1000_ T_ = 20.38) and but a higher sequence conservation value (C_I_ = 0.843) (compared with C_L_ = 0.836 in landraces and C_T_ = 0.813 in teosintes). The inbred lines also exhibited a lower nucleotide diversity throughout the entire genomic region of *ZmNAC087* compared with the other two populations (Fig. 5B). These data collectively suggest that *ZmNAC087* has undergone selection during maize domestication and evolution, showing significant divergence in the promoter region, second intron, and downstream region. The entire *ZmNAC087* genomic sequence was further subjected to neutrality tests and no significant differences in Tajima's D and Fu's and Li's values were detected based on individual populations (Fig. 5A). However, Tajima's D values of the promoter and downstream regions exhibited significant difference across three populations (Table 1). Notably, Fu and Li's values were negative across all tested regions (Table 1). Taken together, these analyses indicate the possibility of *ZmNAC087* being subjected to selection during the process of maize domestication.

To further investigate the potential selection of *ZmNAC087*, a coalescent simulation was employed that incorporated the maize domestication bottleneck [48, 49]. This simulation estimated the likelihood of the observed reduction in genetic diversity in landraces and maize inbred lines compared to teosintes. Deviations from the expected outcomes under a neutral domestication bottleneck were observed in all tested regions of *ZmNAC087* except for the 5'-UTR (Fig. 5D). These findings suggest that the reduced genetic diversity in these regions in landraces and maize inbred lines compared with teosintes is not solely due to the maize domestication bottleneck. Considering that SNP-472 explained the largest proportion of the observed phenotypic variation, we analyzed its allele frequencies across the three populations. Frequency of the SNP-472^A^ allele increased from 12.50% in the teosinte population to 36.62% in landraces, and further rose to 69.64% in inbred lines (Fig. 5C). These findings suggest that *ZmNAC087* underwent artificial selection during maize domestication and improvement.

## Discussion

Soil salinity is a well-known constraint to crop growth and productivity, leading to the prevalence of saline farmland. Maize exhibits moderate responsiveness to salt stress, posing challenges in saline environments [50–52]. Understanding the genetic mechanisms underlying salt tolerance and identifying relevant alleles/genes are pressing needs for crop improvement. Plant roots are key in perceiving and responding to soil salinity and are therefore highly susceptible to salt stress [25, 53]. Salinity significantly hampers the growth of maize seedling roots [54]. Consequently, developing resilient root systems in crops to thrive in saline soils holds great promise for improving water and nutrient uptake and ensuring sustainable yield production.

Considering that salt tolerance is a complex genetic trait, harnessing the rich diversity of naturally available germplasm represents a cost-effective approach to enhancing salt tolerance in modern cultivars [55]. Previous studies have identified salt-tolerant variants in natural crop populations, presenting an opportunity to leverage these variants for enhancing the tolerance of in crops [56–58]. In this study, we conducted high-resolution QTL mapping by employing a panel of 140,714 dense and high-quality SNPs that spanned the 2.3-Gb maize genome with an average interval of 50 kb between SNPs. We identified a total of 69 markers that were significantly associated with TRL (root length) under both normal and salt-stress conditions and the majority of these markers were mapped at the single-gene level. Surprisingly, we were unable to identify co-localized loci/genes with previously published studies [50, 59–61]. This discrepancy may be attributed to differences in association populations, traits investigated, and developmental stages for salinity tolerance evaluation, highlighting the complexity of salt tolerance in maize. Our study has identified several promising genes that warrant further investigation in the context of plant salt responses and the breeding of salt-tolerant maize varieties. Specifically, a SNP residing in the *ZmNAC087* gene was found to be associated with the salt stress response. We analyzed the 1-Mb genomic region flanking this SNP and found that no polymorphisms in LD with this SNP were significantly associated with maize TRL under salt stress, suggesting that *ZmNAC087* may be the only causal gene. It is worth noting that *ZmNAC087* is a nuclear-localized NAC transcription factor with preferential expression in the LR primordium and stele of the PR. These results collectively support the notion that *ZmNAC087* plays a key role in modulating salt tolerance and root growth.

Despite the cloning of many transcription factors (TFs) necessary for root survival in saline environments [62–64], the contribution of allelic sequence variations to salt stress-induced root phenotypic differences remains largely unknown. To date, only a few salt-tolerant QTLs have been identified through association mapping [18, 50, 59, 65]. In this study, we used association mapping to pinpoint the genetic variations linked to maize salt tolerance and identified significant SNPs/Indels in *ZmNAC087* promoter that significantly correlated with the root length (TRL) in salt-stressed maize seedlings. This enabled the classification of *ZmNAC087* variants into two major haplotypes, Hap1 and Hap2, which led to differential *ZmNAC087* expression under salt stress and confer different levels of salt-tolerance in maize inbred lines. The *ZmNAC087* ^Hap2^ allele that emerged from this study will therefore be a potential target for improving salt tolerance in maize.

LUC activity was greater when driven by *ZmNAC087* ^Hap2^ promoter compared with that driven by *ZmNAC087* ^Hap1^, providing solid evidence that genetic variations in *ZmNAC087* promoter are responsible for differential *ZmNAC087* expression. The analysis of 39 maize inbred lines that carried either Hap1 or Hap2 under normal and salt-stressed conditions substantiated the association between *ZmNAC087* expression and salt-regulated TRL, further establishing a link between genetic variations in *ZmNAC087* promoter and salt tolerance in maize. The NAC TFs have been reported to contribute to plant stress tolerance in Arabidopsis, and ANAC087, which is the closest homolog of *ZmNAC087*, was found to target the columella root cap and regulate programmed cell death [66]. Therefore, *ZmNAC087* may play a key role in promoting root development by enhancing water and nutrient availability under salt stress. These findings are in line with our GWAS results and demonstrate the effectiveness of using GWAS to identify natural variations associated with salt tolerance in maize. Importantly, our study provides additional evidence for the influence of natural variations in the promoter region on gene function, which is in agreement with previous findings on the impact of natural variations in *ZmSULTR3;4* and *ZmCBL8* promoters on maize salt tolerance [13, 65]. However, the high degree of LD among the identified polymorphisms hampers the identification of the causal site in Hap2 responsible for increased *ZmNAC087* expression in maize varieties with this haplotype. Future nucleotide substitution analysis will help to pinpoint the causal variation responsible for increased *ZmNAC087* expression as well as enhanced root development and salt tolerance.

Genetic diversity provides valuable resources for improving crop yield and stress resistance [55, 67, 68]. Several stress-related genes, such as *ZmSULTR3:4*, *bZIP68*, and *KRN4*, have experienced strong selection during the genetic improvement of maize [65, 69, 70]. Here, we observed a consistent decline in genetic diversity of *ZmNAC087* in maize inbred lines compared with landraces and teosintes. The promoter and downstream region of *ZmNAC087* exhibited a more pronounced reduction in nucleotide polymorphisms, indicating a relatively higher level of selective pressure in these regions. The result of the neutrality tests indicate that *ZmNAC087* was most likely selected during maize domestication. The pronounced disparities observed in the tested regions of *ZmNAC087* from the anticipated outcomes under a neutral domestication bottleneck suggest that the genetic diversity of *ZmNAC087* may have been substantially influenced by selection. Consistent with this finding, our sequence analyses showed that the favorable allele *ZmNAC087* ^SNP−472A^ occurred at a low frequency in teosintes but its frequency gradually increased in landraces and inbred lines during domestication and maize improvement. This result is consistent with recent research demonstrating the accumulation of favorable alleles in modern inbred lines and their hybrids [71]. Our study suggests that the *ZmNAC087* ^SNP−472A^ allele may not have been fully utilized in modern maize breeding and can therefore be employed to enhance salt tolerance in maize. Further investigation is required to explore the biological function and regulatory network of *ZmNAC087* through the utilization of genetic methodologies, including overexpression and CRISPR-Cas9.These efforts will facilitate the effective utilization of the *ZmNAC087* genetic variations for enhancing stress tolerance.

## Conclusions

The results of our GWAS study provide a list of potential candidates for further investigation into the genetic and molecular mechanisms responsible for the variability of salt-regulated root growth in maize seedlings and highlight the significant involvement of *ZmNAC087*. The natural variation identified in *ZmNAC087*, particularly the ZmNAC087^Hap2^ allele, which has undergone selection during maize domestication and improvement, holds great promise for enhancing maize salt tolerance and represents an important target for the development of salt-tolerant maize germplasm.

## Methods

### Methods

#### Plant cultivation and phenotypic evaluation of the maize association panel

In this study, we evaluated the total root length (TRL) of 280 maize inbred lines during the seedling stage. The plants were cultivated within a controlled greenhouse environment at Yangzhou University during the period spanning from September to October 2017. For plant cultivation, we implemented a paper roll system following the methodology outlined by Li et al. (2021) [31]. In short, seeds of similar sizes underwent surface sterilization by soaking in a 10% hydrogen peroxide solution for 20 min, followed by two rinses employing distilled water. Subsequently, the seeds were immersed in a concentrated calcium sulfate solution for a duration of 6 h. The treated seeds were then positioned vertically inside black containers filled with 7.5 L of nutrient solution [31]. The nutrient solution composition has been documented in a study conducted by Li et al. (2021) [31]. The nourishing solution was replenished every other day while cultivating the seedlings. After five days, the nutrient solution was augmented with or without a concentration of 100 mmol·L^–1^ NaCl. The study utilized a fully randomized layout and was replicated two times. After seven days of NaCl treatment (when the maize seedlings reached the three- to four-leaf stage), the roots were collected and scanned to obtain images for TRL analysis, utilizing the WinRHIZO software (Pro 2004b, Canada).

### GWAS

A GWAS was performed on a naturally occurring population comprising 280 maize inbred lines [72]. The association panel was sequenced using the genotyping-by-sequencing (GBS) technique. Tassel 5.0 was utilized for principal component and kinship analysis, employing a set of 140,714 single nucleotide polymorphisms (SNPs) with a minor allele frequency (MAF) of at least 0.05 and a missing rate below 0.2. Using the ADMIXTURE software (version 1.3), the population structure matrix (Q) was computed by utilizing the initial five principal components. The TASSEL software utilized the centered IBS approach to compute the kinship matrix (K) and assess the genetic relatedness between different accessions. The GWAS analysis was conducted using a conventional mixed linear model (MLM) that integrated both K and Q [73]. A significance threshold of 1/effective number of SNPs (1.96 × 10^–5^) was used to identify associations between markers and traits. The percentage of phenotypic variance explained by each significant SNP was estimated as described previously [74]. Linear models of the form *Y* = *αX* + *βP* + *ε* were constructed, where *X* represented the SNP genotype, *P* represented the first two principal components, *α* represented the SNP effect, *β* represented the *PC* effects, and *ε* represented the error term.

### DNA extraction, *ZmNAC087* resequencing, and association analysis

The CTAB method was employed for the extraction of DNA from the juvenile leaves of maize seedlings. The data obtained from the TRL analysis of the 280 maize inbred lines was utilized for the purpose of analyzing associations. The *ZmNAC087* gene sequence data was acquired through the utilization of a targeted sequence capture technology offered by BGI Life Tech Co., China, in accordance with the protocols provided by the manufacturer (Roche/NimbleGen) [75]. The DNA samples were broken down using sonication, and then adaptors were introduced to the resultant fragments. Fragments of the desired size were obtained through PCR amplification, followed by purification and hybridization to the capture array at 42.0 °C using the buffer provided by the manufacturer. The array underwent two washes at a temperature of 47.5 °C and three washes at the temperature of the surrounding environment. After purification with the DNA Clean & Concentrator-25 Kit, the fragments obtained were subjected to quality assessment using Bioanalyzer (Agilent) before sequencing on the Illumina platform. Eliminated adapters and reads of inferior quality, then aligned the clean reads to the B73 reference genome (AGPv3.31) using Burrow-Wheeler Aligner (BWA) employing the parameters 'mem -t 4 -k 32 -M' [76]. The GATK 4.0 [77] was utilized for the execution of gene sequence conversion and variant calling. The MAFFT software was used to perform a multiple sequence alignment of *ZmNAC087*, and apparent mismatches were corrected manually using BioEdit [78, 79]. Nucleotide polymorphisms with a MAF ≥ 0.05 were identified using TASSEL 5.0 [73], and their association with TRL was evaluated using the MLM model. The significance threshold was set at a *p*-value of 0.01/n, where n refers to the number of SNPs and InDels.

### Genetic diversity analyses, neutrality tests, and coalescent simulations

The MAFFT software was used to align the *ZmNAC087* genome sequence across all tested lines, and any apparent mismatches were corrected manually using BioEdit. The B73 sequence (AGPv3.31) was used as a reference to identify gene features such as untranslated regions, exons, and introns. Genetic diversity and neutral evolution of the populations were analyzed using DNASP v5 software [80]. To assess the extent of genetic variation in the population, the values of π and θ were utilized as parameters. Specifically, π symbolizes the mean count of dissimilarities in genetic building blocks among two separate sequences, while θ is derived from the overall count of varying positions and modified to account for the size of the sample [81]. The π and θ values for *ZmNAC087* were analyzed utilizing a window size of 100 bp and a step size of 25 bp. Neutrality tests, namely Tajima's D and Fu and Li's tests, were performed utilizing DNASP v5 [82, 83]. In accordance with the methodology outlined by Tian et al. (2009) [49], coalescent simulations were performed to integrate the domestication bottleneck into the analyzed regions of *ZmNAC087* [84–86]*.* The population recombination parameters and population mutation were estimated based on the data obtained from teosintes. Coalescent simulations were performed using Hudson's ms program with 10,000 replicates per simulation [87].

### Phylogenetic analysis of *ZmNAC087*

Amino acid sequences of NAC TF family members in Arabidopsis, rice, and maize were downloaded from Phytozome database version 10.0. In order to align amino acid sequences of NAC TF members, Clustal X 1.83 software was used with default parameters. MEGA 5.0 was utilized to construct a phylogenetic tree by employing the neighbor-joining method with the alignment results. The construction parameters included pairwise deletion, uniform rates, Poisson correction, and bootstrap analysis with 1,000 replicates.

### Tissue localization analysis of ZmNAC087

The in situ hybridization procedures were conducted at Servicebio Biotechnology Co. LTD., China. The root tissues were preserved in a FAA solution at a temperature of 4 °C subsequent to their collection from seedlings that had been cultivated in a hydroponic solution for a duration of 7 days. Paraffin embedding was conducted, and the roots were sectioned using a sliding slicer and placed on polylysine-coated slides. After deparaffinization, digestion with proteinase K, and dehydration in gradient ethanol, the samples were hybridized with the antisense probe. The slides underwent a washing process and were subsequently incubated with anti-digoxigenin-AP Fab fragments to facilitate immunological detection, employing the NBT/BCIP method.

### Subcellular localization of ZmNAC087

For subcellular localization analysis, the *pGreenII-Ubi:GFP* vector was utilized to clone the complete coding sequence of *ZmNAC087* through the *BamH* I site. Protoplasts from the mesophyll of maize were obtained from B73 leaves that were etiolated on the 14th day after sowing [88, 89]. PEG-mediated transformation was used to introduce the *Ubi:ZmNAC087-GFP* and *H2B-mCherry* plasmids into protoplasts, and *H2B-mCherry* was used to visualize nuclear structure [47]. Transformed protoplasts were observed using a Zeiss LSM710 confocal microscope.

### Transcriptional activation analysis of ZmNAC087

In order to examine the transactivation capability of *ZmNAC087* in yeast, we cloned the complete coding sequence (1–348 aa, ZmNAC087), the N-terminal region (1–148 aa, ZmNAC087-N), and the C-terminal region (149–348 aa, ZmNAC087-C) into the pGBKT7 vector (Clontech) separately. Transactivation activity was analyzed as previously described [90]. The obtained plasmids were introduced into yeast strain AH109 and subsequently cultured on synthetic dextrose minimal medium lacking tryptophan (SD/-Trp). The yeast concentration was adjusted to an OD600 value of 0.1, and sequential dilutions were made at ratios of 1/10, 1/100, and 1/1000. The yeast samples were then cultured on synthetic dextrose minimal medium lacking tryptophan, histidine, and adenine (SD/-Trp-His-Ade) to assess viability. The plates were subjected to incubation at a temperature of 30 °C for a duration of 3 days prior to capturing photographs. The primer sequences used in the study are listed in Table S3.

### *ZmNAC087* promoter activity assay

The dual luciferase promoter activity assays were conducted following a previously described method [65]. Briefly, ~ 1.4-kb fragments of the *ZmNAC087* promoter were cloned into the *pGreen II-0800-LUC* vector. Maize mesophyll protoplasts were transfected as described above. The *35S* promoter was utilized to drive the expression of Renilla luciferase (REN) as an internal control for evaluating transfection efficiency. Luciferase (LUC) activity was measured using the dual-luciferase reporter assay system (Promega) following the guidelines provided by the manufacturer.

### RNA extraction and qRT-PCR analysis

After 8 days of hydroponic growth, root samples from the inbred lines were subjected to a two-day treatment with or without 100 mM NaCl, similar to the phenotypic screening of TRL in the natural population described earlier. Subsequently, samples were collected for quantitative analysis of *ZmNAC087*. The Trizon Reagent was utilized to extract total RNA from fresh roots. Then, a quantity of 2 μg of total RNA underwent treatment with RNase-free DNase I prior to the synthesis of the first strand complementary DNA, which was accomplished through the utilization of M-MLV reverse transcriptase (Promega). The qRT-PCR analyses were conducted on the Roche Light Cycler 480 real-time PCR system using SYBR Premix Ex TaqII (Takara). The internal reference *ZmUbi2* was employed for data normalization, and the calculation of gene expression levels was conducted utilizing the 2^–ΔΔCT^ method.

### Data analysis

The statistical significance of the variance in TRL, LUC activity, and gene expression levels between the control and treatment groups was assessed using the unpaired two-tailed Student's *t*-test. Furthermore, the statistical methods of Tajima's D, Fu and Li's test, and coalescent simulations were employed to ascertain the potential selection of *ZmNAC087*. Symbols * and ** are used to denote statistical significance at the significance levels of *p* < 0.05 and *p* < 0.01, respectively.

### Research involving plants

The experimental research and materials utilized in this study comply with applicable institutional, national, and international guidelines and legislation. Prior permission was obtained for the collection of maize seeds used in the present study.

### Supplementary Information

**Additional file 1:** **Table S1.** SNPs and candidate genes associated with TRL under normal and salt-stress conditions.

**Additional file 2:** **Table S2.** Allelic variation of the *ZmNAC087* genomic region and their association with total root length (TRL) in response to salt stress.

**Additional file 3:** **Table S3.** Digoxigenin (DIG)-labeled probe and PCR primer sequence used in the present research.

**Additional file 4:** **Fig. S1.** The GWAS was conducted using total root length (TRL) under normal growth conditions. The horizontal dashed line represented significance threshold (-log_10_*P* = 4.71).

**Additional file 5:** **Fig. S2. **Phylogenic analysis of NAC TF family in Arabidopsis, rice and maize. The phylogenetic tree was generated using MEGA-X software with the Poisson model and 1000 bootstrap values. The numbers of the branches are the bootstrap values from 1,000 replicates.

## Data Availability

The data sets that support the results presented in this article, along with its supplementary files, are included in the publication. All raw reads have been deposited into the NCBI Sequence Read Archive (SRA, http://www.ncbi.nlm.nih.gov/sra/) and can be accessed under accession number PRJNA683126.

## Abbreviations

CTAB: Cetyltrimethylammonium bromide
GFP: Green fluorescent protein
GWAS: Genome-wide association study
MLM: Mixed linear model
Hap: Haplotype
SNP: Single nucleotide polymorphism
InDel: Insertion-deletion mutations
LD: Linkage disequilibrium
LUC: Luciferase
MAF: Minor allele frequency
PEG: Polyethylene glycol
Ren: Renilla Luciferase
TRL: Total root length
UTR: Untranslated region
QTL: Quantitative trait loci
