# Supplementary material for: Natural variation in ZmNAC087 contributes to total root length regulation in maize seedlings under salt stress
Source: BMC Plant Biol. 2023 Aug 14;23:392. doi: 10.1186/s12870-023-04393-7 (PMC10424409; doi:10.1186/s12870-023-04393-7)
Supplement: Supplementary file 4 — Additional file 4: Fig. S1. The GWAS was conducted using total root length (TRL) under normal growth conditions. The horizontal dashed line represented significance threshold (-log10P = 4.71). [file 12870_2023_4393_MOESM4_ESM.docx]

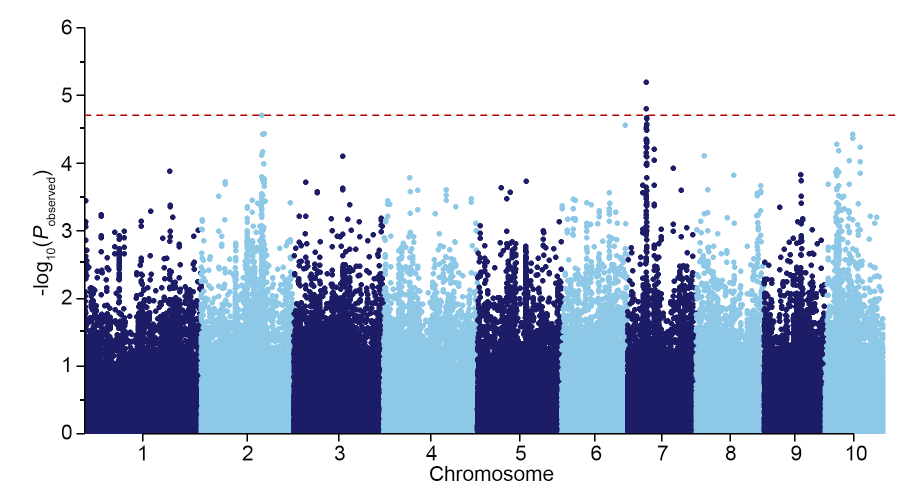


**Fig.S****1** The GWAS was conducted using total root length (TRL) under normal growth conditions. The horizontal dashed line represented significance threshold (-log_10_ *P* = 4.71).
