# Supplementary material for: Natural variation in ZmNAC087 contributes to total root length regulation in maize seedlings under salt stress
Source: BMC Plant Biol. 2023 Aug 14;23:392. doi: 10.1186/s12870-023-04393-7 (PMC10424409; doi:10.1186/s12870-023-04393-7)
Supplement: Supplementary file 5 — Additional file 5: Fig. S2. Phylogenic analysis of NAC TF family in Arabidopsis, rice and maize. The phylogenetic tree was generated using MEGA-X software with the Poisson model and 1000 bootstrap values. The numbers of the branches are the bootstrap values from 1,000 replicates. [file 12870_2023_4393_MOESM5_ESM.docx]

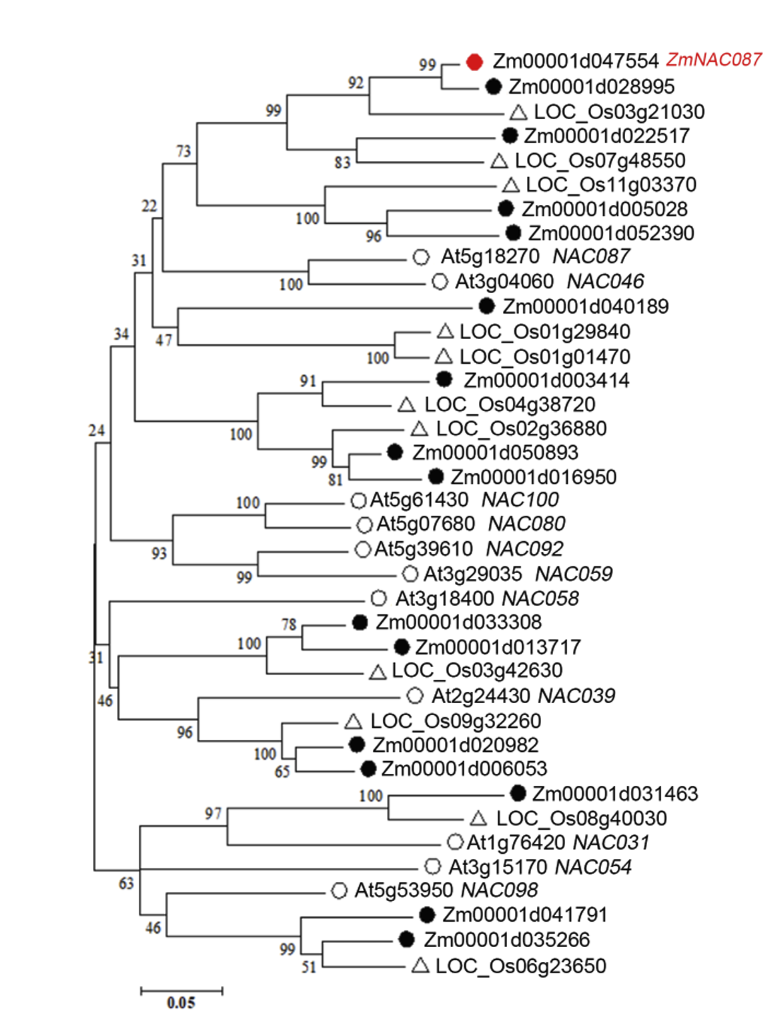


**Fig.S2** Phylogenic analysis of NAC TF family in Arabidopsis, rice and maize. The phylogenetic tree was generated using MEGA-X software with the Poisson model and 1000 bootstrap values. The numbers of the branches are the bootstrap values from 1,000 replicates.
